# Antisymmetry of oceanic eddies across the Kuroshio over a shelfbreak

**DOI:** 10.1038/s41598-017-07059-1

**Published:** 2017-07-28

**Authors:** Yu Liu, Changming Dong, Xiaohui Liu, Jihai Dong

**Affiliations:** 1 Marine Science College, Nanjing University of Information Science & Technology, Nanjing, 210044 China; 2 Jiangsu Engineering Technology Research Center of Marine Environment Detection, Nanjing, 210044 China; 3 Oceanic Modeling and Observation Laboratory, Nanjing University of Information Science & Technology, Nanjing, 210044 China; 4 Department of Atmospheric and Oceanic Sciences, University of California, Los Angeles, CA 90095 USA; 5 State Key Laboratory of Satellite Oceanic Environment and Dynamics SIO/SOA, Hangzhou, 310012 China

## Abstract

From the analysis of oceanic eddies detected in the drifter trajectories of the Global Drifter Program (GDP) data set, it was found that oceanic eddies are asymmetrically distributed across the Kuroshio in the East China Sea: predominant cyclonic (anticyclonic) eddies are on the western (eastern) sides of Kuroshio. This distribution is confirmed by high-resolution numerical modeling output as well. Most of these eddies are 5~20 km in radius, less than the local first baroclinic deformation radius, thus categorized as submesoscale. The generation mechanism of these submesoscale eddies is speculated to be related to the horizontal velocity shear of the Kuroshio when it flows northeastward along the shelf break in the East China Sea. The budget analysis of eddy kinetic energy shows that both the horizontal shear and vertical buoyancy flux are important energy sources for eddy generation on the two sides of Kuroshio axis. The finding highlights the unique feature of oceanic eddies along the western boundary currents.

## Introduction

**Figure 1:**
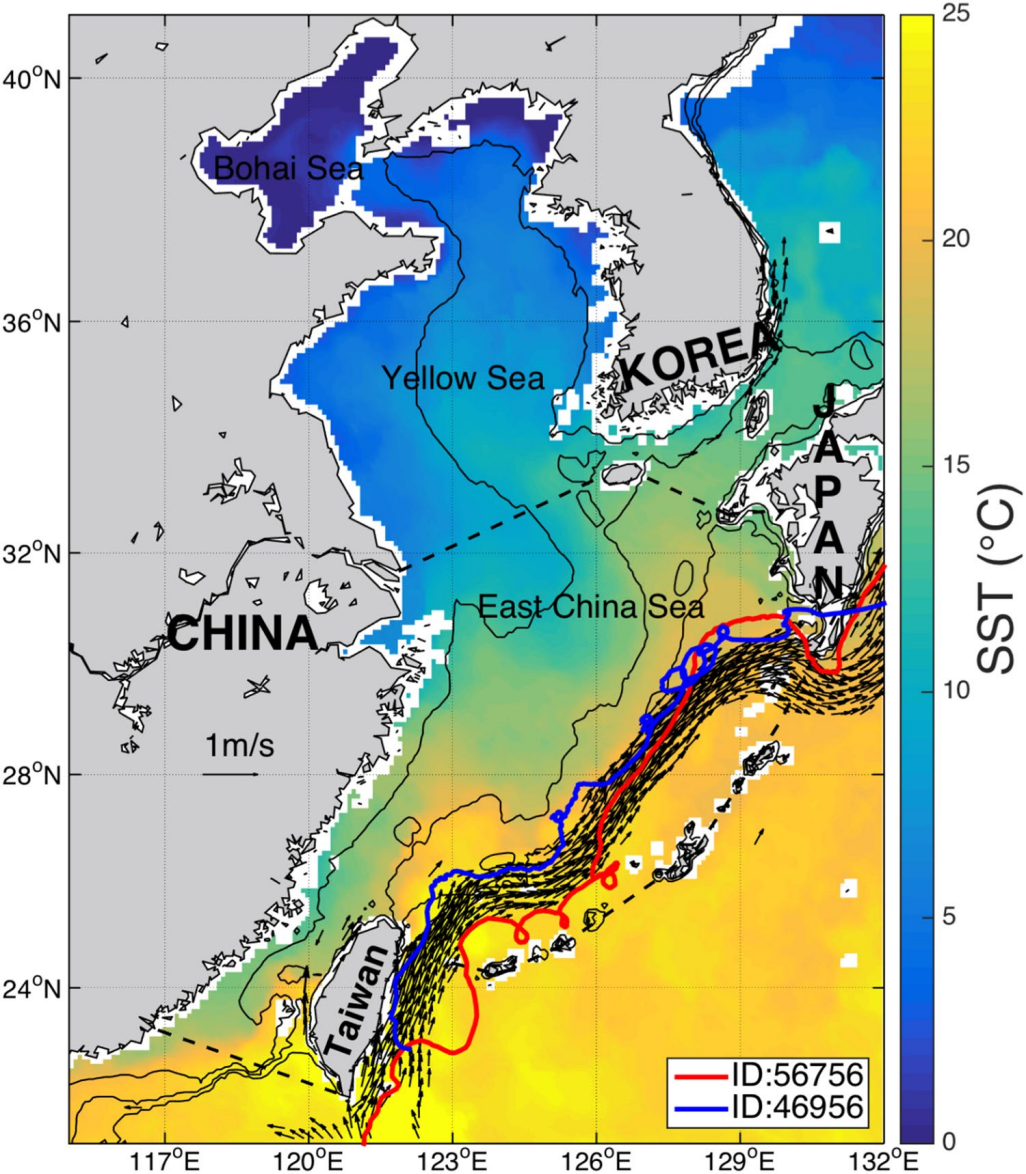
The study area: East China Sea area with boundaries marked out by the dashed line. The northwest boundary is from Qidong to Jeju Island. The northeast boundary is from Jeju Island to Fukue-jima to Nagasaki. The south boundary is from Nan’ao Island to the southernmost of the Taiwan Island. The east boundary is from Kyushu and along the Ryukyu Islands (Osumi Shoto, Amami Islands, Okinawa Shoto) to Taiwan Island. Examples of cyclonic and anticyclonic eddies along the climatological Kuroshio Current (the black arrows, >50 cm/s) averaged within 0.2°*0.2° bin from the 2000–2015 Global Drifter Program (GDP) trajectory data set. Blue line is the trajectory of drifter with ID46956, and red line is the trajectory of drifter with ID56756. The sea surface temperature (SST) front can be clearly seen near the 50, 100 and 200 m isobaths (thin black lines). The SST data is come from the 1^st^-Jan-2013 RSS Microwave-IR (MW-IR) Optimal Interpolated SST (version 4.0) data set. Figure is plotted using MATLAB R2014b (http://www.mathworks.com/) with M_Map (a mapping package, http://www.eos.ubc.ca/~rich/map.html).

The Kuroshio is a well-known energetic western boundary current in the northwest Pacific Ocean (see Fig. 1). From both observational analysis^1^ and statistical detection results from various satellite data sets^2, 3^, abundant eddies occur along the Kuroshio path. Most studies of such eddies (in terms of generation or interaction) are related to the Kuroshio meandering path in some specific regions, e.g., in the northern Okinawa Trough^4, 5^, between the Tokara Strait and south of Kyushu^6^, in the northeast of Taiwan Island^7^, and in the Luzon Strait^8^.

In the East China Sea, the Kuroshio current diligently follows the continental shelf/slope break, which is significantly different than its meandering path elsewhere. Its axis is stable and flows along isobaths. Additionally, the seasonal variability is very small. This part of the Kuroshio is denoted “the main part of the Kuroshio in the East China Sea”^9^ (MPK for short) and can be thought as a stable parallel flow. Because the mean horizontal shear (a source for barotropic instability) is an important dynamic mechanism of eddy generation, we speculate that the MPK should produce mesoscale or sub-mesoscale eddies along its two sides and with eddy size constrained by the width of MPK. These eddies are not easily visualized in the low-resolution satellite images^2, 3^, so the drifter data and high-resolution model output are employed to detect these eddies.

## Results

The climatological Kuroshio with speed larger than 50 cm s^−1^ is shown in bold black arrows in the area east to the mainland of China and west to the Ryukyu Islands (Fig. 1). It is derived from 2000–2013 GDP drifter trajectory data. The blue line is the trajectory of drifter ID46956 with cyclonic loops; the red line is the trajectory of drifter ID56756 with anticyclonic loops. It can be clearly seen that a cyclonic eddy and an anticyclonic eddy are present on the western and eastern sides of the Kuroshio, respectively.

**Figure 2:**
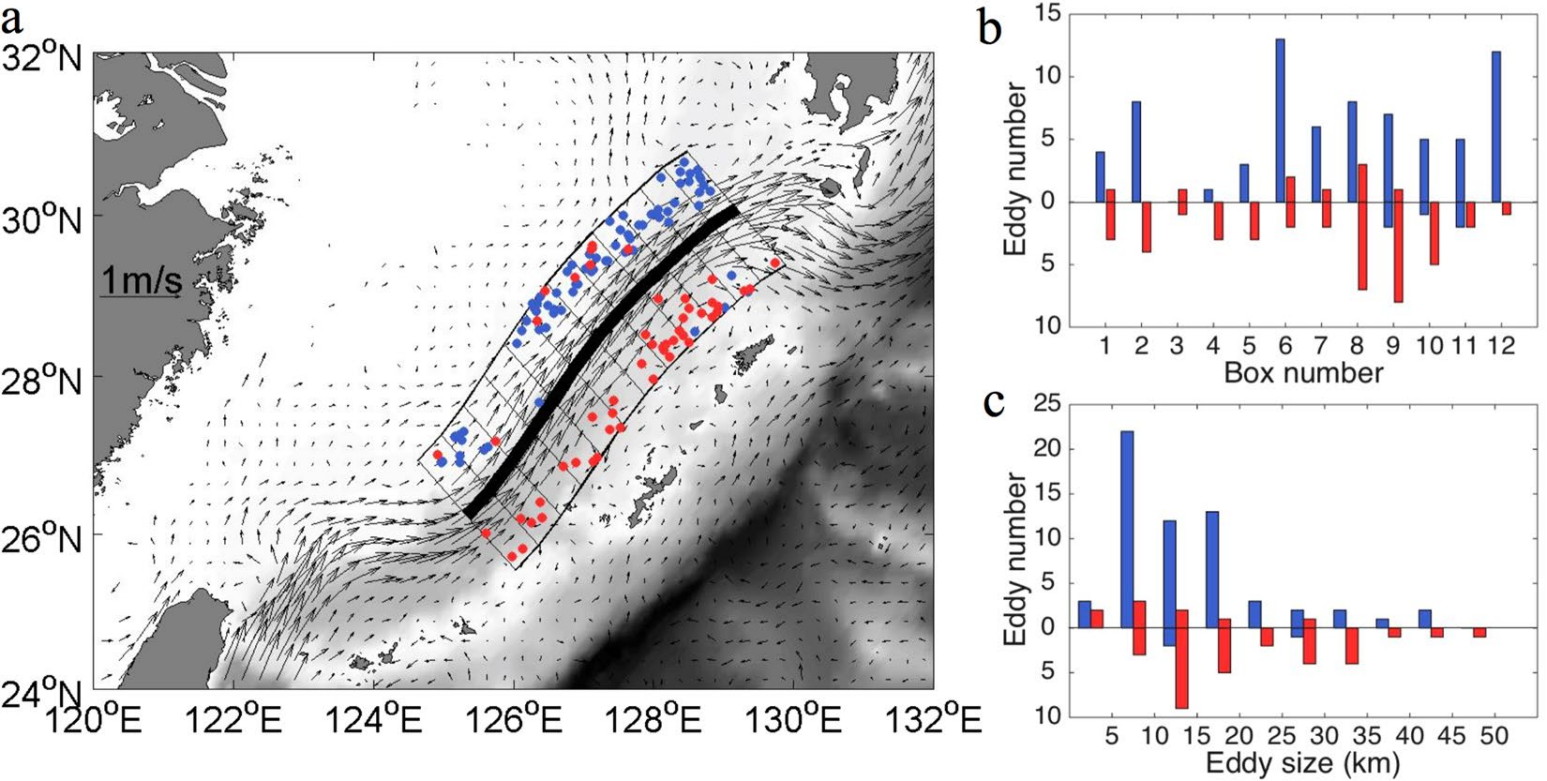
(**a**) The distribution of eddies detected from the GDP trajectories using Dong *et al*.^10^ eddy detection scheme. Blue and red dots denote cyclonic and anticyclonic eddies, respectively. More cyclonic (anticyclonic) eddies are detected on the western (eastern) side of the Kuroshio. The black arrows are the climatological velocity averaged within 0.2°*0.2° bin from the 2000–2015 GDP trajectory data set. Along the climatological Kuroshio axis (the thick black line), 24 bins with about 70 km in width by 20 km in length are used for eddy statistics. (**b**) Eddy number within each bin. The zero line indicates ‘along the Kuroshio path’, and positive (negative) value corresponds to ‘eddy on the western (eastern) side of Kuroshio axis’. Blue (red) bars are cyclonic (anticyclonic) eddy numbers. (**c**) Eddy size histogram showing most eddies are sub-mesoscale with about 5–20 km in radius. Figures are plotted using MATLAB R2014b (http://www.mathworks.com/) with M_Map (a mapping package, http://www.eos.ubc.ca/~rich/map.html).

Using an eddy detection scheme (ref. 10) with the Lagrangian data, cyclonic and anticyclonic loops (eddies) are detected. They are denoted by blue dots (cyclonic) and red dots (anticyclonic), respectively (Fig. 2a). The thick black line is the main axis of the Kuroshio and defined by the maximum mean velocity values. Grey arrows outside of the Kuroshio region denote that drifter records are insufficient. There are clearly more cyclonic (anticyclonic) eddies are on the western (eastern) side of the Kuroshio.

Along the MPK axis (the solid line), the local region is divided into 12*2 bins which extend out by about 70 km on both sides of the MPK axis. The distance between two adjacent lines perpendicular to the MPK axis is about 20 km. We determined that number of eddies and their size for these bins along the Kuroshio path. In Fig. 2b, the zero line denotes the Kuroshio climatological axis. The bars above (below) the zero line are the numbers of eddies on the western (eastern) side of the Kuroshio. The blue (red) dots mark the mean position of the cyclonic (anticyclonic) eddies. Totally, there are 72 cyclonic eddies and nine anticyclonic eddies on the western side of the MPK; and there are five cyclonic eddies and 41 anticyclonic eddies on the eastern side of the MPK. There are significantly more cyclonic (anticyclonic) eddies in each bin on the western (eastern) side of the MPK, and fewer cyclonic (anticyclonic) eddies on the opposite side. The results show that the characteristics of eddies’ distribution in the MPK, namely, polarity-antisymmetric distribution. More cyclonic (anticyclonic) polarity eddies are generated on the western (eastern) side of the Kuroshio. In the eddy size histogram (Fig. 2c), radii of most eddies are about 5–20 km, which is smaller than the first baroclinic deformation radius 50 km in the area^11^.

**Figure 3:**
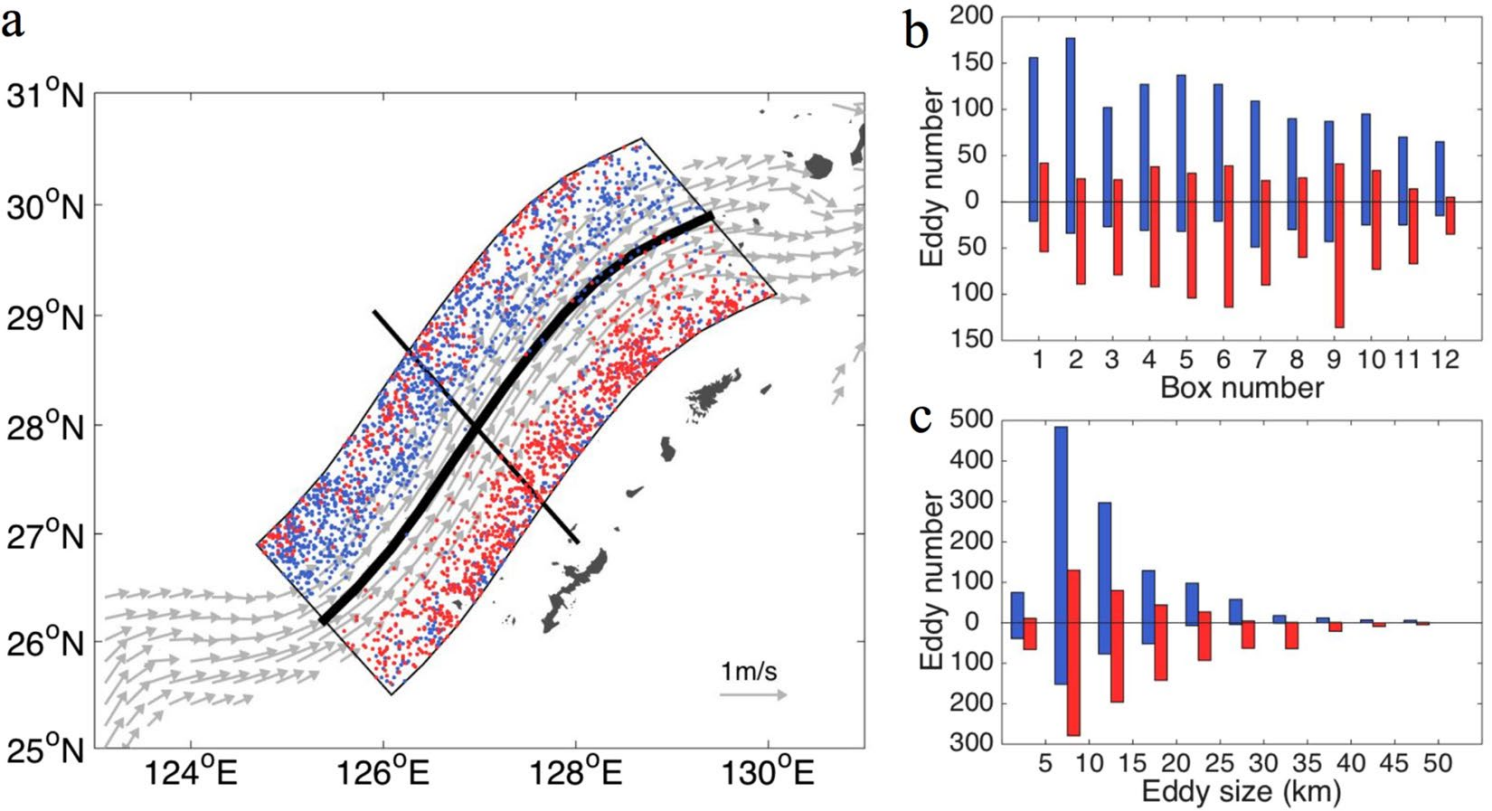
(**a**) Distribution of eddies detected from high-resolution model using Necioli *et al*.’s^12^ eddy detection scheme. Blue and red dots denote cyclonic and anticyclonic eddies, respectively. The Kuroshio velocity with the speed of larger than 40 cm/s is shown by the grey arrows. The climatological Kuroshio axis (thick black line) was determined by the maximum speed. The black line perpendicular to the Kuroshio axis is used for deriving horizontal current profiles along the direction of the Kuroshio axis. (**b**) Eddy number within each bin in Fig. 2. Same as Fig. 2, except for eddies detected from high resolution model outputs. (**c**) Eddy size histogram. Figures are plotted using MATLAB R2014b (http://www.mathworks.com/) with M_Map (a mapping package, http://www.eos.ubc.ca/~rich/map.html).

To further confirm the above results based on the limited drifter data, we employed an Eulerian method^12^ to detect eddies from high-resolution modeling output. The results are presented in Fig. 3a. Along the MPK axis region (thick black line) extending out 70 km on both sides, the total 1342 cyclonic eddies and 342 anticyclonic eddies are found on the western side of the MPK; the total 353 cyclonic eddies and 993 anticyclonic eddies on the eastern side of the MPK. Their centers are marked by blue (cyclonic) and red (anticyclonic) dots. The mean currents larger than 50 cms^−1^ are marked by gray arrows. The results again confirm that the polarity-antisymmetric distribution of oceanic eddies exist across the MPK.

Repeating the same processes used for the drifter data, we obtained statistics for the eddy numbers in 12*2 bins along the Kuroshio path (bins are not shown in Fig. 3a). In the histogram (Fig. 3b), the zero line again denotes the Kuroshio climatological axis. The bars above (below) the zero line stand for numbers of eddies on the western(eastern) side of the Kuroshio. The blue (red) dots are the centers positions of cyclonic (anticyclonic) eddies. There are about one hundred cyclonic (anticyclonic) eddies in the western (eastern) side of the MPK, and fewer cyclonic (anticyclonic) eddies on the opposite side. Again, the results show that the characteristics of eddies’ distribution in the MPK, namely, polarity-antisymmetric distribution. In the eddy size histogram (Fig. 3c), we can see that most eddies are sub-mesoscale and their radii are about 5–20 km. Both data sets from different sources, drifters and model output, show similar results. Additionally, the histogram of normalized vorticity shows the skewness of vorticity that the cyclonic vorticity is stronger than the anticyclonic vorticity (Supplementary Fig. S1).

Using two different methods—Lagrangian (drifter’s trajectories) and Euler (model’s velocity fields) schemes, we reveal the statistical characteristics of eddy distribution along the main part of the Kuroshio in the East China Sea: polarity-antisymmetric distribution. Most eddies are sub-mesoscale and their radii are peak at 5–20 km. And the cyclonic vorticity is stronger than the anticyclonic vorticity.

**Figure 4:**
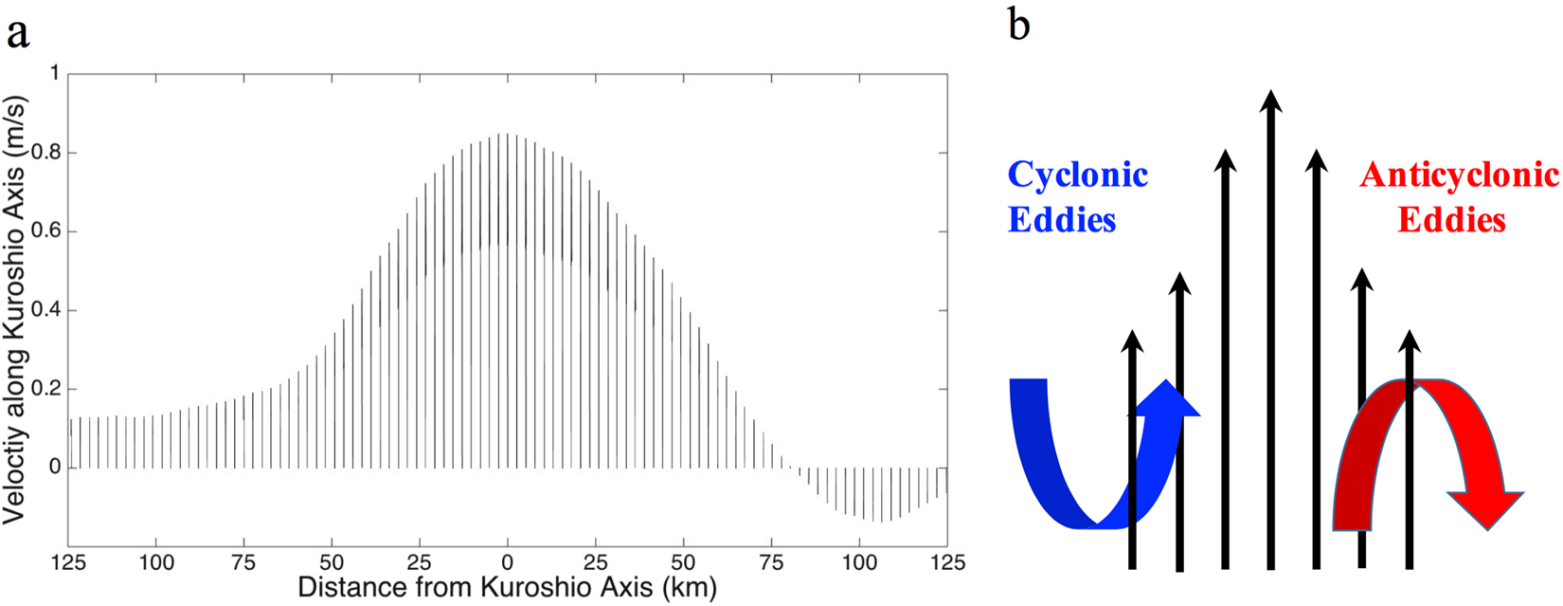
(**a**) Climatological velocity vector profiles along the thick black line (as x-axis) in Fig. 3a. It shows the character of horizontal flow parallel to the Kuroshio axis. (**b**) Schematic diagram of eddies shed from a parallel flow.

For better understanding the phenomenon, we randomly select a line (Fig. 3a) perpendicular to the MPK. The interpolated velocity profiles cross the line show normal distribution in Fig. 4a. On the western (eastern) side of the axis is the positive (negative) horizontal velocity shear, which easily leads to the formation of cyclonic (anticyclonic) eddies, see the schematic figure: Fig. 4b. Potential vorticity (PV) analysis shows that there exists a change in sign of the horizontal gradient of PV along the lines perpendicular to the MPK, see Methods and Supplementary Fig. S2. According to the quasigeostrophic theory (ref. 13), this is the necessary condition for a normal-mode instability with a parallel shear flow: barotropic instability or baroclinic instability when the dominant sign change is mainly due to horizontal shear or vertical shear. So, we speculate that the horizontal shear instability and baroclinic instability could be responsible for the generation of polarity-antisymmetry of oceanic submesoscale eddies across the Kuroshio in the East China Sea.

Based on the eddy kinetic energy (EKE) analysis, the eddy generation mechanism is primarily^14, 15^ due to a barotropic instability (HRS > 0), a vertical shear instability (VRS > 0) or a baroclinic instability (VBF > 0), see Methods for the definition and Supplementary Fig. S3 for the annual mean results. The HRS is bigger than the VRS for about one order of magnitude (absolute value) along the MPK. The conversion from mean kinetic energy to eddy kinetic energy is mainly due to the horizontal shear production term. The HRS is positive on the northwest part on the western side and most areas on the eastern side of the Kuroshio axis, which implies the HRS is important for the eddy generation. And the VBF is positive in most areas on the western side of the Kuroshio axis. We integrated the HRS, VRS and VBF along the Kuroshio axis from south to north. The Supplementary Fig. S4 presents the variations of HRS, VRS and VBF across the Kuroshio axis. The annual mean shows that the VBF acts as an energy source and HRS acts as an energy sink for the eddy energy on the western side of the Kuroshio axis, and the HRS acts as a main energy source for the eddy energy on the eastern side.

The EKE budget has strong seasonality. The monthly mean of the EKE analysis is shown in Supplementary Fig. S5. The positive HRS can be clearly seen in January, April, June and December. Therefore, the HRS also make significant contribution to the eddy generation on the western side of Kuroshio axis with the spatial and temporal variations. Moreover, the previous PV analysis also support this conclusion. So, both the HRS and VBF are important energy sources for eddy generation on the both sides of Kuroshio axis.

To diagnose the scale of energy conversion, we select the December data and a relatively smaller region along the Kuroshio axis (longitude [127°E~129°E] and latitude [26°N~29°N]) to do the wavenumber spectral analyses (see Supplementary Fig. S6). For the relative small scale k = 10^−4^/m (10 km), the VBF power spectra is larger than the HRS’s. And for the relative larger scale k = 10^−5^/m (100 km), the HRS power spectra is larger than the VBF’s. It shows that the VBF may play a more important role in the generation of the smaller scale eddies.

It should be noted that ECS have unique features: 1) wide shelf which allows the eddy development on the western side of the Kuroshio. 2) the presence of the Ryuku Islands blocks the influence from open ocean, which is different from the other west boundary currents (e.g. Gulf Stream and East Australia Current). Such antisymmetric phenomenon (polarity-antisymmetry of oceanic submesoscale eddies) and dynamic environment (antisymmetric eddy generation mechanisms) could play an important role in eddy-induced mass and heat transport across the ECS continental shelf.

## Methods

### Data

The drifter data and high-resolution model output are employed in the study to detect eddies. The historical surface drifter data collected by the Global Drifter Program (GDP; ref. 16) are downloaded from http://www.aoml.noaa.gov/phod/dac/dacdata.html. Within the study area (24°–32°N and 120°–132°E), 1592 drifters are available from Jul. 1988 to Jun. 2014. However, only 1379 drifters with more than 40 records (longer than 10 days) are used for the eddy detection.

A high-resolution (1/54°) model output data of (ref. 17) produced by the Regional Oceanic Modeling (ROMS) is also used too. A nested domain is used in the model. The outer domain has a resolution of 1/8° within 110°E–138°E, 15°N–41°N, and the inner domain has a resolution of 1/54° within 120°E–131°E, 24°N–32°N. The inner domain is slight different from that in ref. 17. The ETOPO1 database (http://www.ngdc,noaa.gov/mgg/global/) is interpolated for the bathymetries of the two domains. There are 20 vertical layers in both model domains. For the outer domain, the initial and open boundary conditions are based on the climatological data (for the time period of 2003–2012) extracted from the 1/12° global analysis of Navy Coupled Ocean Data Assimilation (NCODA) and Hybrid coordinate Ocean Model (HYCOM). Both domains are forced at the surface by the seasonally varying climatological fluxes of heat. Buoyancy is from the Comprehensive Ocean-Atmospheric Data Set (COADS). Again different from ref. 17, we use year 2000 QuikSCAT daily wind field for the momentum flux rather than seasonally varying climatological fluxes of momentum from the COADS. The outer domain is first integrated for five years to reach a quasi-steady state, followed by another seven years of integration along with the inner domain, which is started at the end of the fifth year of outer domain. We use the last-year result in this study. For more detailed information and model evaluation, please see ref. 17.

### Eddy Detection Schemes

In this study, we utilize two independent detection methods to study eddies along the MPK. One is Lagrangian eddy detection scheme^10^, and the other is Eulerian eddy detection scheme^12^.

A Lagrangian eddy detection scheme is used to identify loops from the drifters’ trajectories. When a drifter is trapped in an eddy, it makes a loop trajectory. To further confirm loops as eddies, a few criteria are used, e.g. the rotational period must be longer than the local inertial period and shorter than the seasonal scale, and there must be at least two consecutive loops with the same polarity in close proximity. This method was successfully applied to the Kuroshio Extension Region (ref. 10).

An Eulerian eddy detection scheme^12^ is also used to analyze the surface velocity field from a numerical modeling. This is an eddy detection scheme based on the velocity geometry field, which identifies rotation around an eddy center. For further information about this method, please see ref. 12. This method has been successfully applied to the model outputs^12, 18, 19^, sea level anomalies-derived geostrophic velocity anomaly data^20, 21^, and sea surface temperature-derived thermal-wind velocity data^22^.

### PV Analysis

The Ertel potential vorticity (PV) is defined as

1 $${q}_{e}=-\frac{1}{{\rho }_{0}}(f\hat{{\bf{z}}}+\nabla \times {\bf{u}})\cdot \nabla \rho ,$$

where *f* is the Coriolis parameter, $\hat{{\bf{z}}}$ is the vertical coordinate, **u** is the three-dimensional velocity, *ρ* is the density and *ρ*_0_ is the mean reference density.

### Eddy Kinetic Energy Analysis

To understand the eddy generation mechanism, we estimate the EKE sources through the EKE evolution equation, which is expressed as^14, 15^:

2 $$\mathrm{TT}=\mathrm{Trans}+\mathrm{HRS}+\mathrm{VRS}+\mathrm{VBF}+\mathrm{Diffusion},$$

where the time variation term

3 $${\rm{TT}}=\frac{\partial {\rm{EKE}}}{\partial t}=\frac{1}{2}\frac{\partial (\overline{{u^{\prime} }^{2}}+\overline{{v^{\prime} }^{2}})}{\partial t},$$

The transport terms (Trans)

4 $${\rm{T1}}=-\frac{1}{2}\frac{\partial (\overline{{u}_{j}}\overline{{{u^{\prime} }_{i}}^{2}})}{\partial {x}_{j}},$$

5 $${\rm{T2}}=-\frac{1}{2}\frac{\partial (\overline{{u^{\prime} }_{j}{{u^{\prime} }_{i}}^{2}})}{\partial {x}_{j}},$$

6 $${\rm{T3}}=-\frac{1}{{\rho }_{0}}\frac{\partial \overline{{u^{\prime} }_{j}p^{\prime} }}{\partial {x}_{j}},$$

the horizontal shear source

7 $$\mathrm{HRS}=-\overline{{u^{\prime} }_{i}v^{\prime} }\frac{\partial {\bar{u}}_{i}}{\partial y}-\overline{{u^{\prime} }_{i}u^{\prime} }\frac{\partial {\bar{u}}_{i}}{\partial x},$$

the vertical shear source

8 $${\rm{VRS}}=-\overline{{u^{\prime} }_{i}w^{\prime} }\frac{\partial {\bar{u}}_{i}}{\partial z},$$

the vertical buoyancy flux

9 $${\rm{VBF}}=\overline{w^{\prime} b^{\prime} },$$

and the diffusion term

10 $${\rm{VM}}=\overline{{\upsilon ^{\prime} }_{i}{u^{\prime} }_{i}},$$

where *u*_1_ = *u*, *u*_2_ = *v* and *u*_3_ = *w*; *u*_*i*_ (*i* = 1, 2) are the horizontal componets of velocity *u*_*j*_ (*j* = 1, 2, 3); *b* is the buoyancy and *υ*_*i*_ is the vertical mixing. Prime denotes the anomaly with respect to the one-month mean. The over-bar denotes the one-month mean. The horizontal viscosity coefficient is set to zero in the model. The diffusion term is only the vertical mixing.

## Electronic supplementary material

Supplementary Information
