# Supplementary material for: Antisymmetry of oceanic eddies across the Kuroshio over a shelfbreak
Source: Sci Rep. 2017 Jul 28;7:6761. doi: 10.1038/s41598-017-07059-1 (PMC5533746; doi:10.1038/s41598-017-07059-1)
Supplement: Supplementary file 1 — Supplementary Information [file 41598_2017_7059_MOESM1_ESM.pdf]

Supplementary Information for

Antisymmetry of oceanic eddies across the Kuroshio over a shelfbreak

Yu Liu [1,2,3], Changming Dong [1,2,3,4, *], Xiaohui Liu [5], Jihai Dong [1,2,3]

[1] Marine Science College, Nanjing University of Information Science and Technology, Nanjing, 210044, China

[2] Jiangsu Engineering Technology Research Center of Marine Environment Detection, Nanjing, 210044, China.

[3] Oceanic Modeling and Observation Laboratory, Nanjing University of Information Science & Technology, Nanjing, 210044, China.

[4] Department of Atmospheric and Oceanic Sciences, University of California, Los Angeles, CA 90095, USA

[5] State Key Laboratory of Satellite Oceanic Environment and Dynamics SIO/SOA, Hangzhou, 310012, China

* Corresponding author address: Prof. Changming Dong, School of Marine Sciences, Nanjing University of Information Science and Technology, Nanjing, China. Email: cdong@atmos.ucla.edu.

Supplementary figures

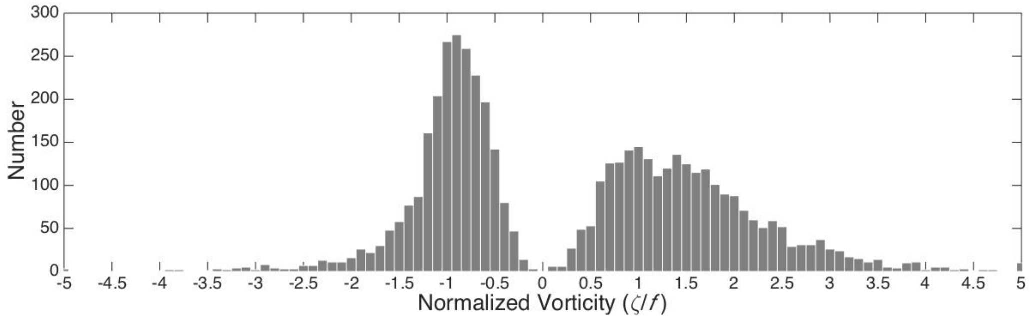

Figure S1 | Histogram of normalized vorticity. Figure is plotted using MATLAB R2014b (http:// www.mathworks.com/).

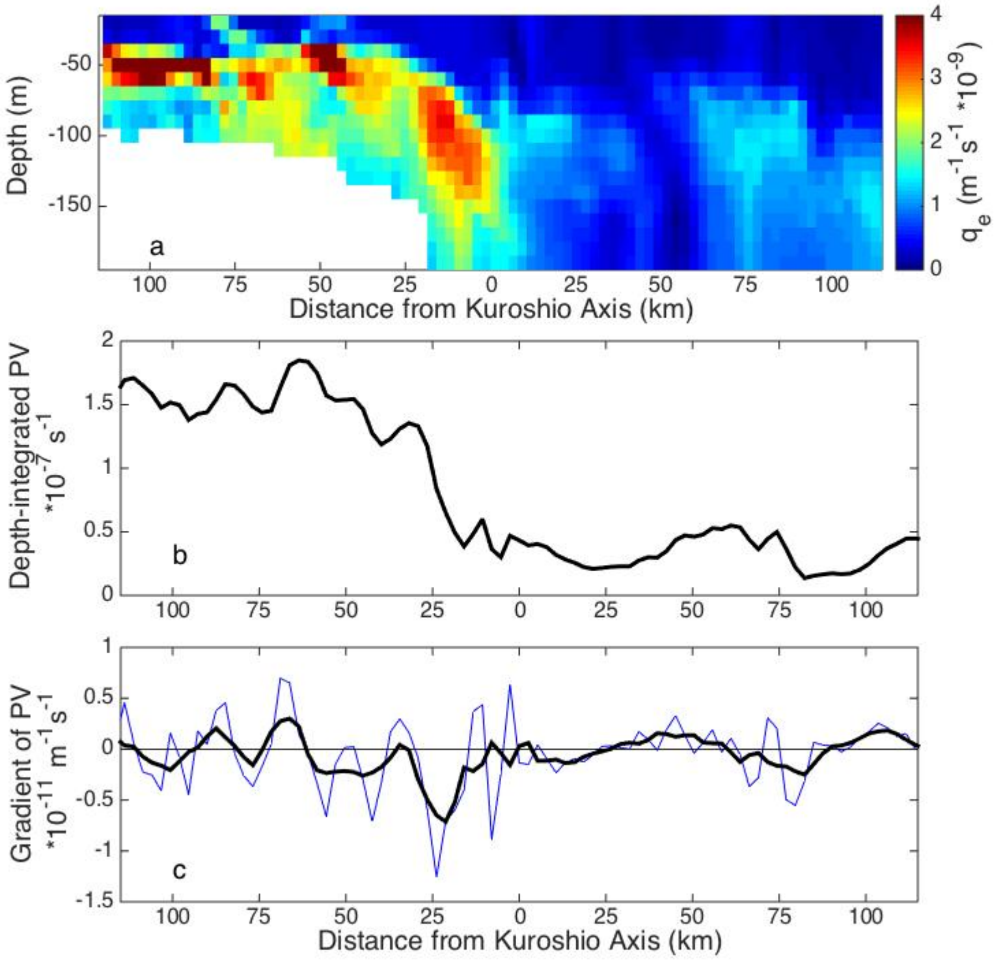

Figure S2 | Along the black line perpendicular to the Kuroshio (See Figure 3). a) instantaneous vertical section of potential vorticity $\mathbf{q_e}$ (unit: 10^{-9} $m^{-1}s^{-1}$); b) instantaneous depth-integrated (upper 80 meters) potential vorticity (unit: 10^{-7} s^{-1}); c) gradient of potential vorticity (unit: 10^{-11} $m^{-1}s^{-1}$), black line is for five points moving smooth. Figures are plotted using MATLAB R2014b (http:// www.mathworks.com/).

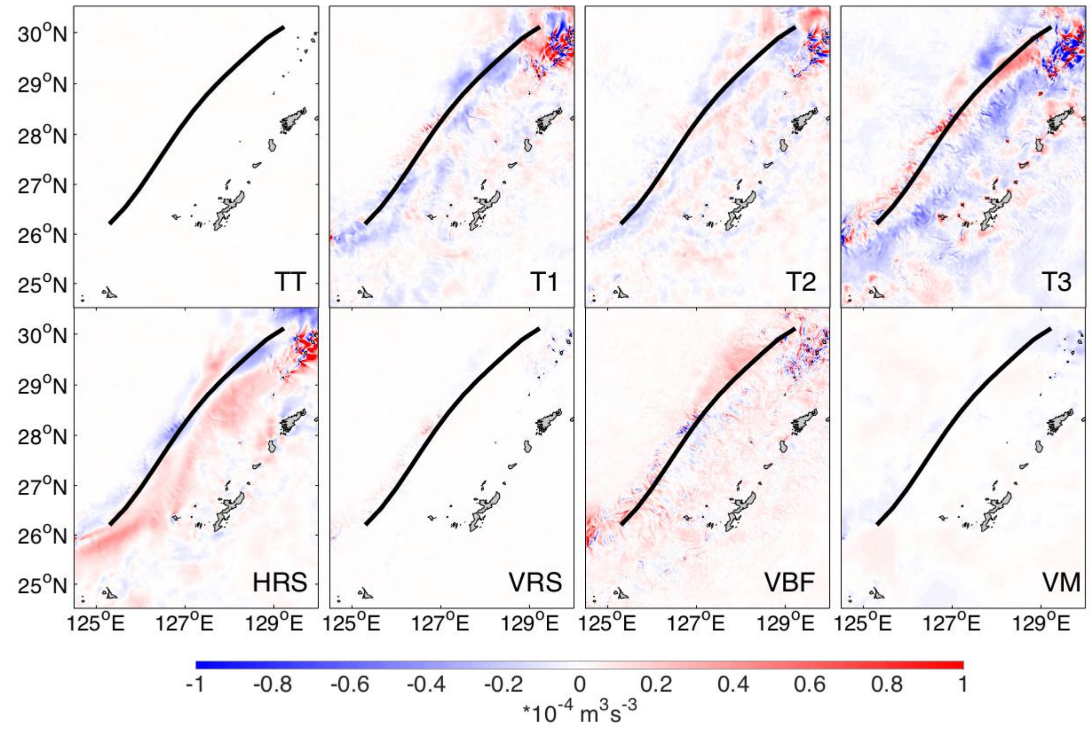

Figure S3 | Annual mean depth-integrated (upper 200 meters) terms of the EKE evolution equation. These terms are time variation term (TT), transports terms (T1, T2 and T3), horizontal shear term (HRS), vertical shear term (VRS), vertical buoyancy flux (VBF) and vertical mixing term (VM). Figures are plotted using MATLAB R2014b (http:// www.mathworks.com/).

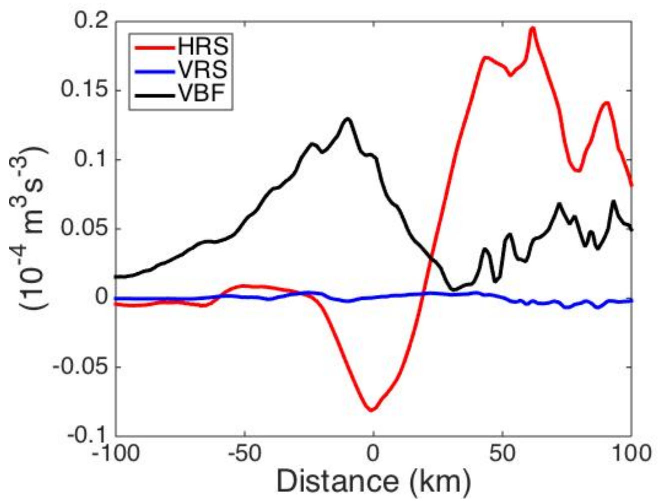

Figure S4 | The annual mean variations of HRS, VRS and VBF across the Kuroshio axis. These terms are integrated along the Kuroshio axis from south to north (black line in the figure S3). Figure is plotted using MATLAB R2014b (http://www.mathworks.com/).

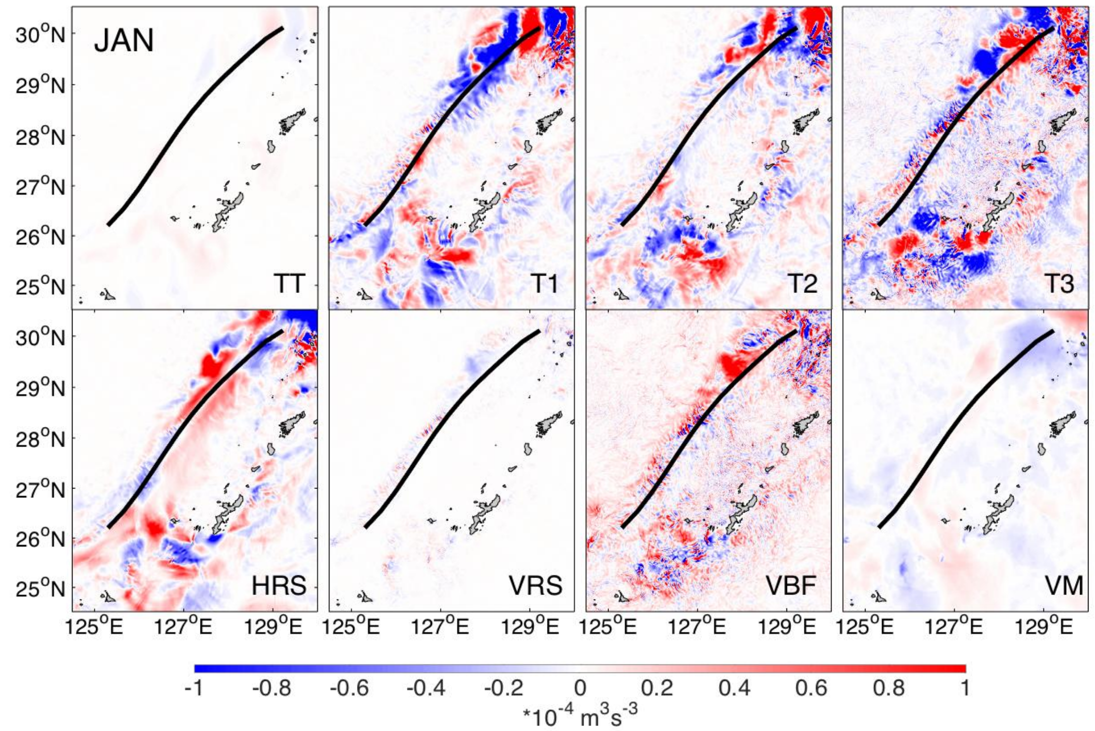
JAN
TT
T1
T2
T3
HRS
VRS
VBF
VM
30°N
29°N
28°N
27°N
26°N
25°N
125°E
127°E
129°E
-1 -0.8 -0.6 -0.4 -0.2 0 0.2 0.4 0.6 0.8 1
*10^{-4} m^{3}s^{-3}

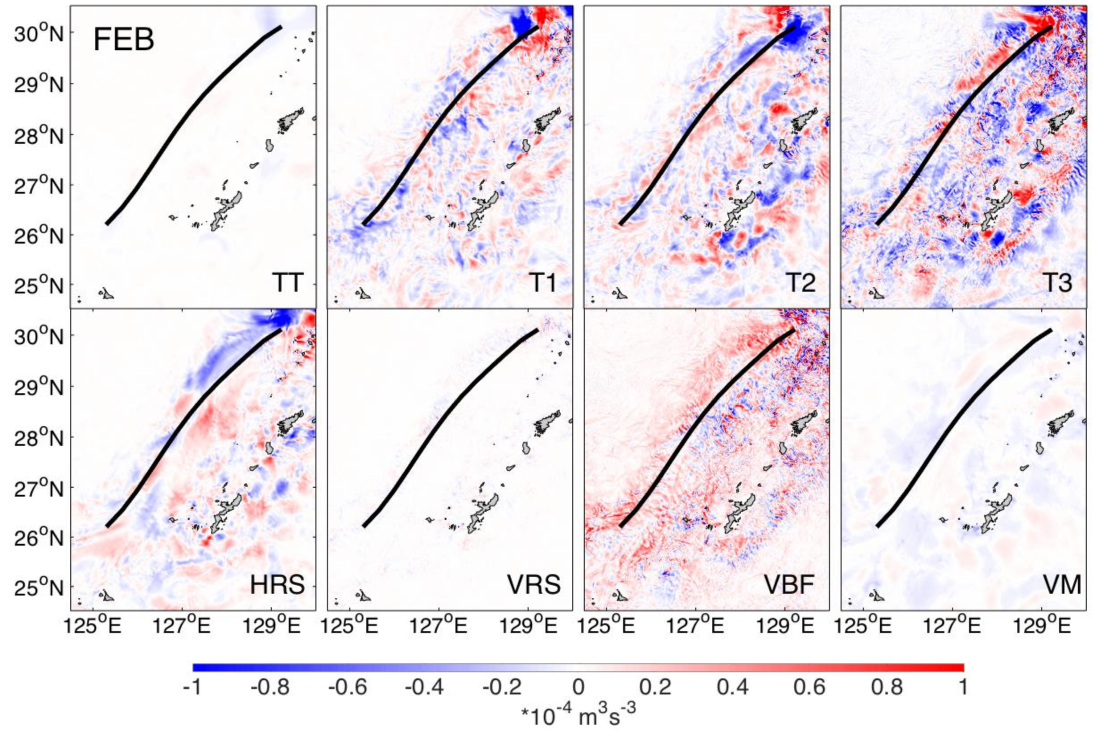
FEB
TT
T1
T2
T3
HRS
VRS
VBF
VM
30°N
29°N
28°N
27°N
26°N
25°N
125°E
127°E
129°E
-1 -0.8 -0.6 -0.4 -0.2 0 0.2 0.4 0.6 0.8 1
*10^{-4} m^{3}s^{-3}

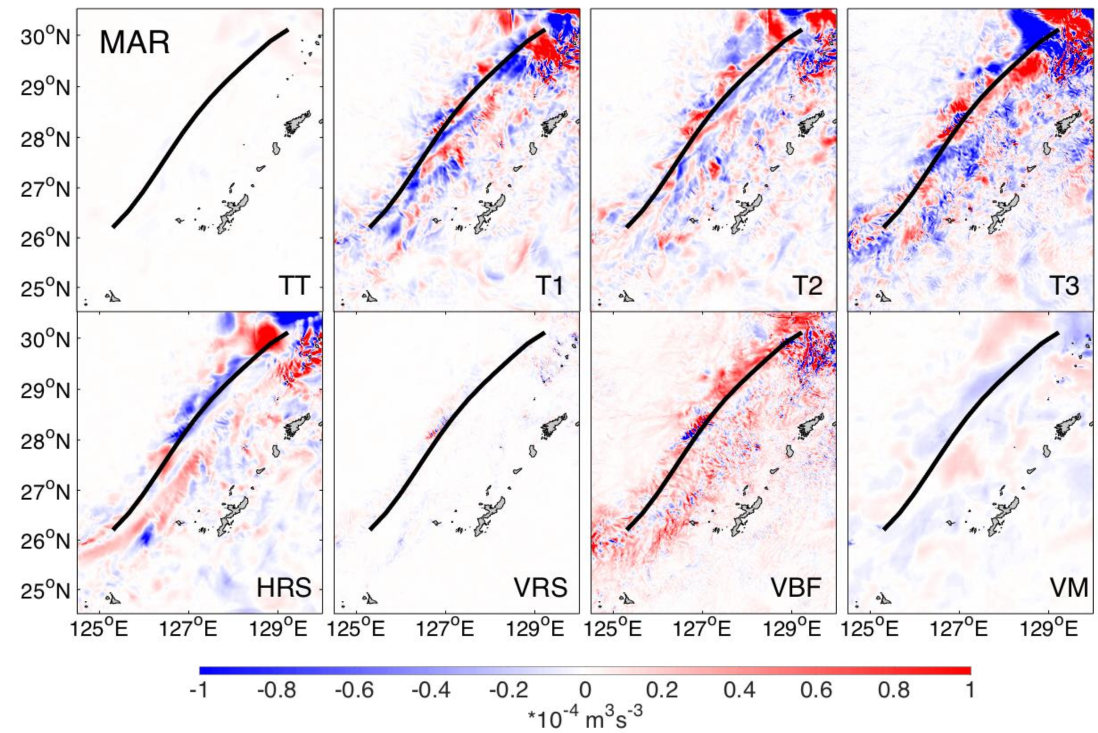

MAR
TT
T1
T2
T3
HRS
VRS
VBF
VM
30°N
29°N
28°N
27°N
26°N
25°N
125°E
127°E
129°E
-1 -0.8 -0.6 -0.4 -0.2 0 0.2 0.4 0.6 0.8 1
*10^-4 m^3s^-3

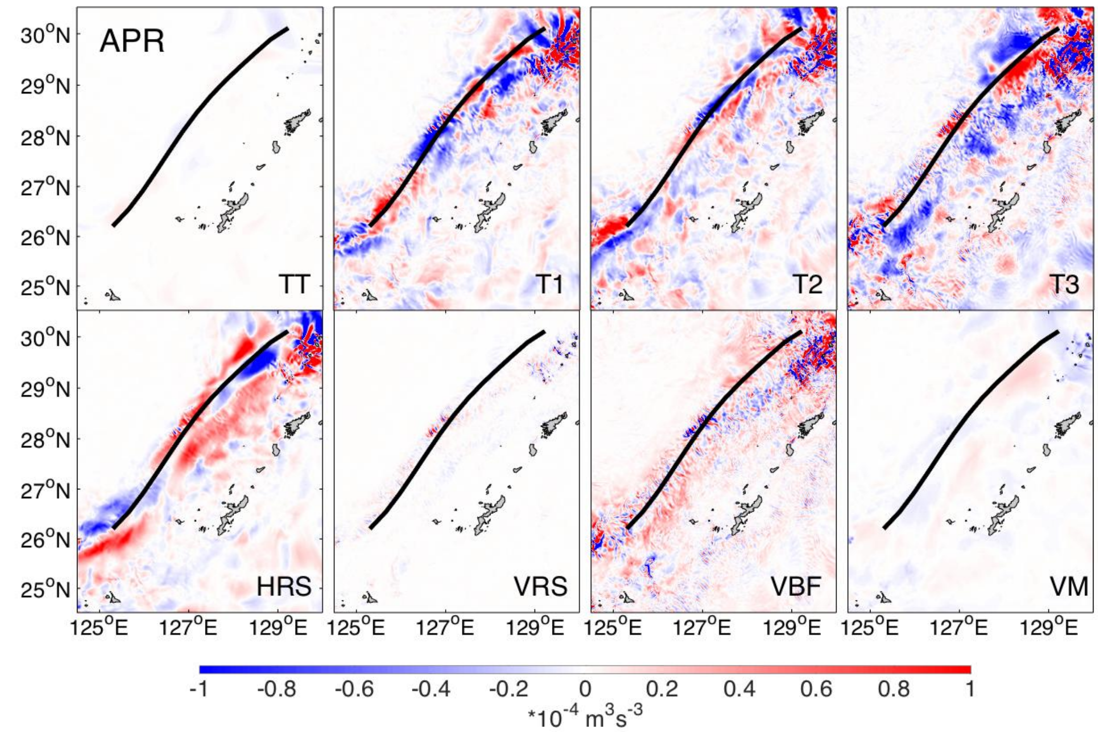

APR
TT
T1
T2
T3
HRS
VRS
VBF
VM
30°N
29°N
28°N
27°N
26°N
25°N
125°E
127°E
129°E
-1 -0.8 -0.6 -0.4 -0.2 0 0.2 0.4 0.6 0.8 1
*10^-4 m^3s^-3

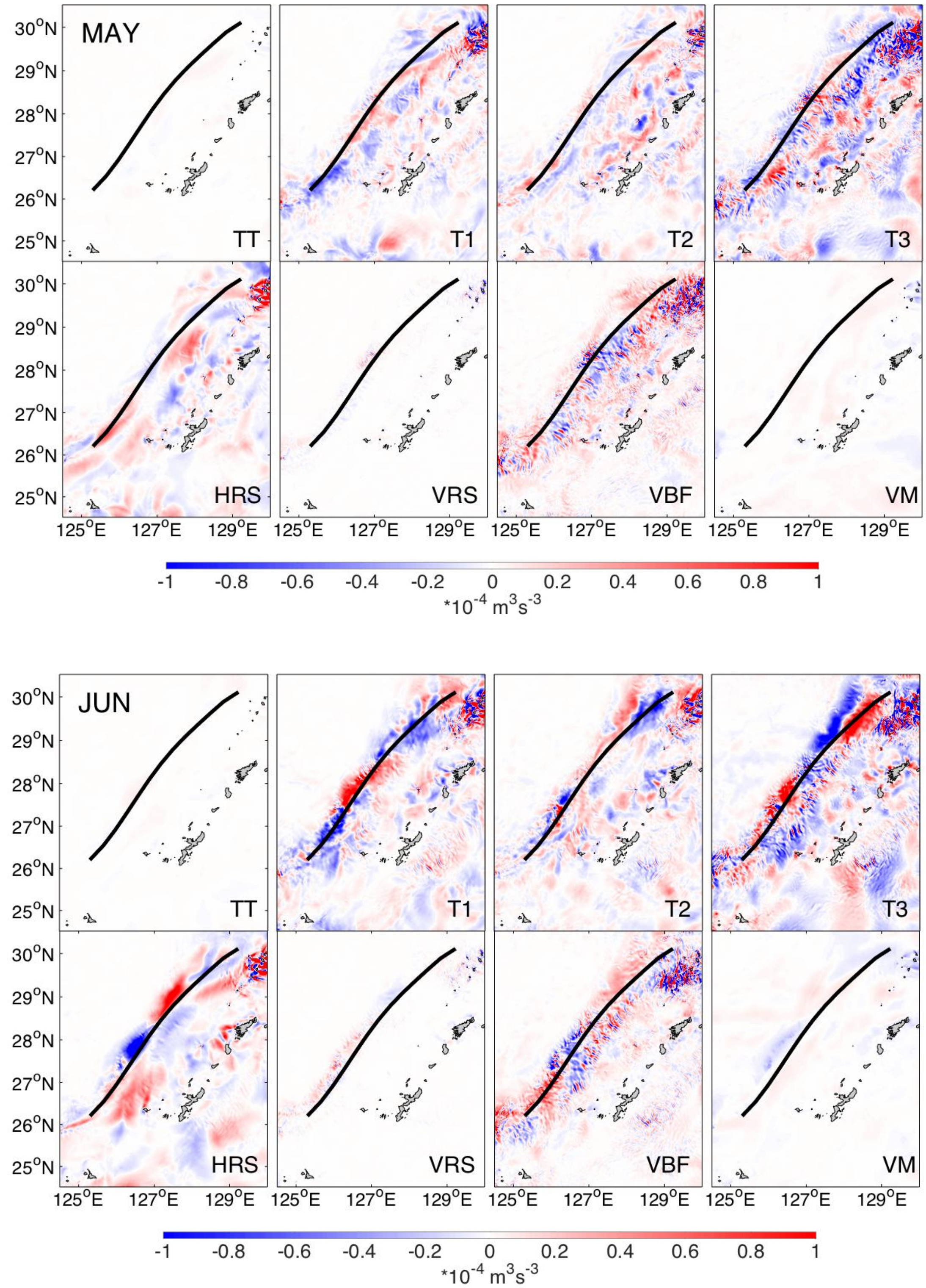
MAY
JUN
TT
T1
T2
T3
HRS
VRS
VBF
VM
30°N
29°N
28°N
27°N
26°N
25°N
125°E
127°E
129°E
-1
-0.8
-0.6
-0.4
-0.2
0
0.2
0.4
0.6
0.8
1
*10^-4 m^3s^-3

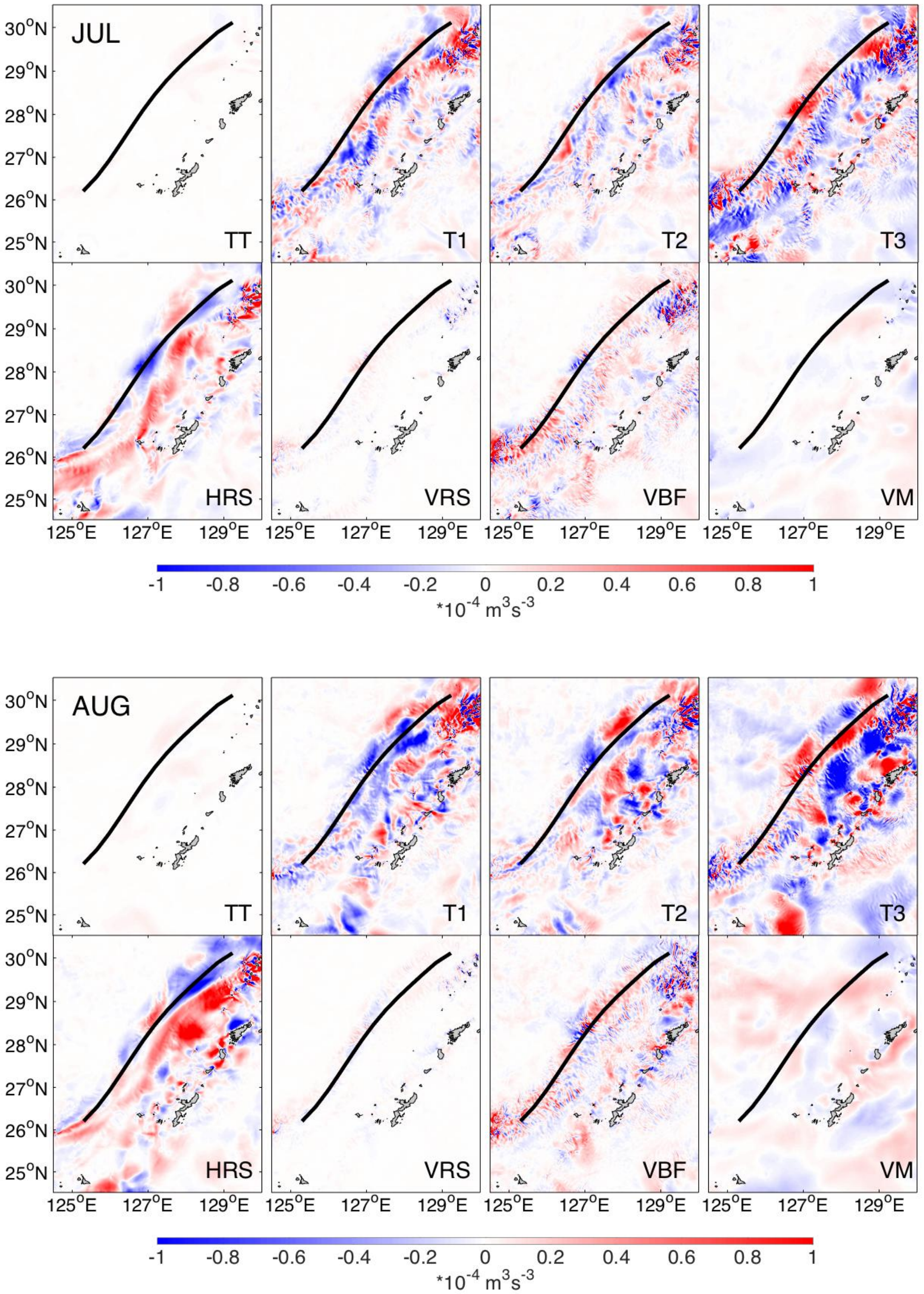
JUL
TT
T1
T2
T3
HRS
VRS
VBF
VM
30°N
29°N
28°N
27°N
26°N
25°N
125°E
127°E
129°E
-1 -0.8 -0.6 -0.4 -0.2 0 0.2 0.4 0.6 0.8 1
*10^-4 m^3s^-3
AUG
TT
T1
T2
T3
HRS
VRS
VBF
VM
30°N
29°N
28°N
27°N
26°N
25°N
125°E
127°E
129°E
-1 -0.8 -0.6 -0.4 -0.2 0 0.2 0.4 0.6 0.8 1
*10^-4 m^3s^-3

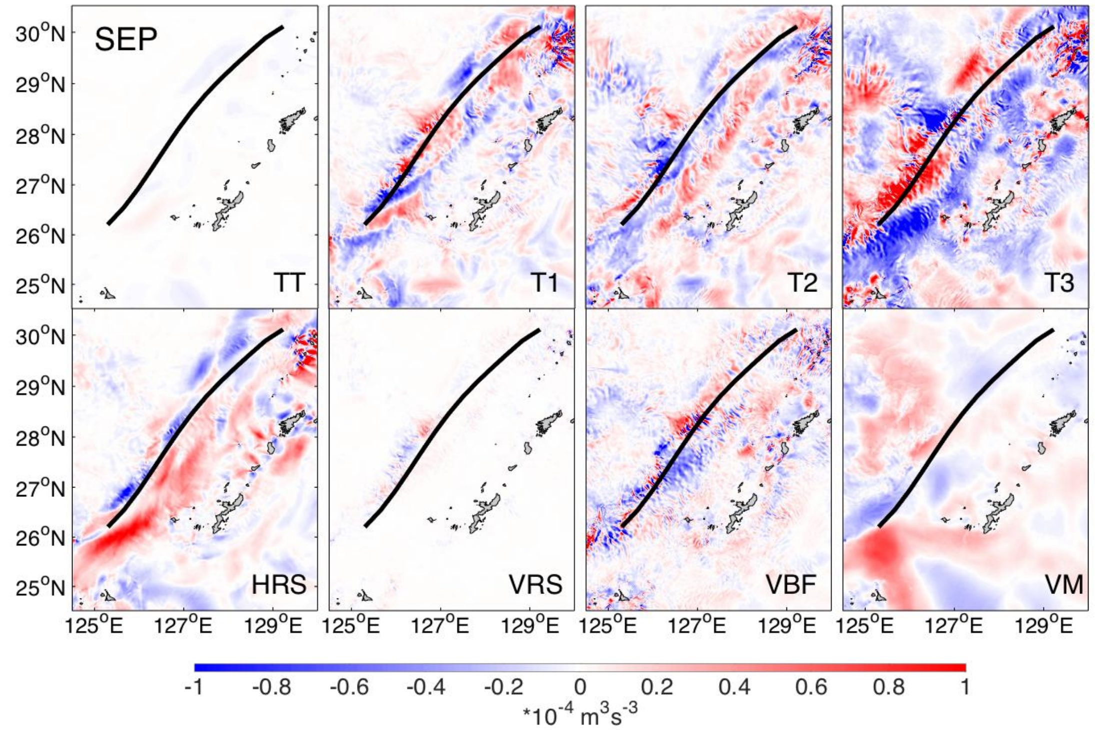
SEP
TT
T1
T2
T3
HRS
VRS
VBF
VM
30°N
29°N
28°N
27°N
26°N
25°N
125°E
127°E
129°E
-1
-0.8
-0.6
-0.4
-0.2
0
0.2
0.4
0.6
0.8
1
*10^-4 m^3s^-3

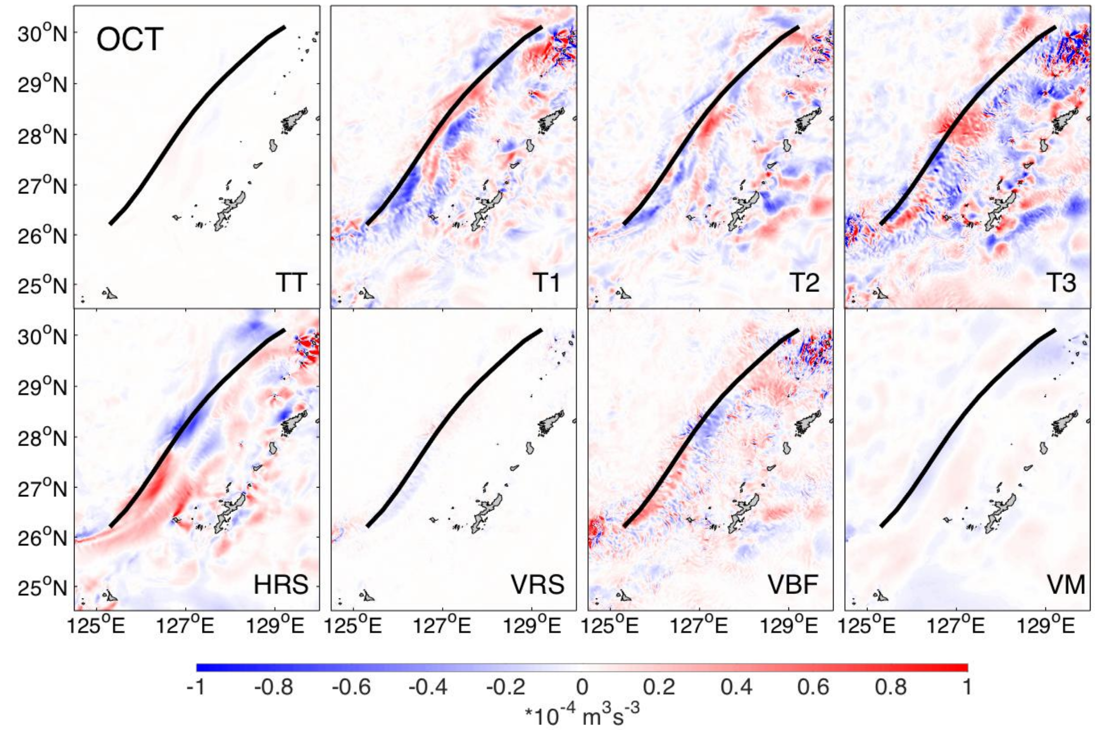
OCT
TT
T1
T2
T3
HRS
VRS
VBF
VM
30°N
29°N
28°N
27°N
26°N
25°N
125°E
127°E
129°E
-1
-0.8
-0.6
-0.4
-0.2
0
0.2
0.4
0.6
0.8
1
*10^-4 m^3s^-3

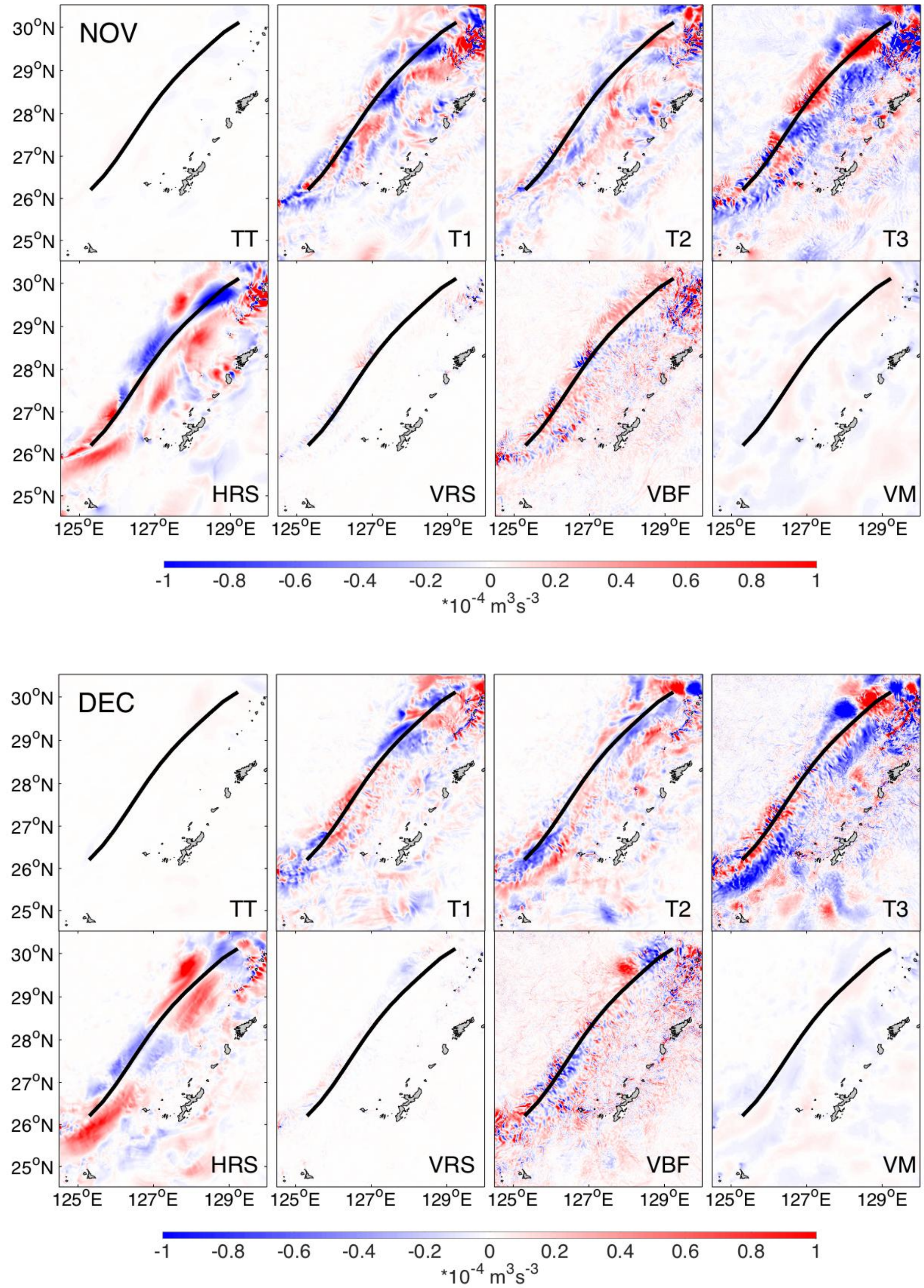

Figure S5 | Monthly mean depth-integrated (upper 200 meters) terms of the EKE evolution equation. These terms are time variation term (TT), transports terms (T1, T2 and T3), horizontal shear term (HRS), vertical shear term (VRS), vertical buoyancy flux (VBF) and vertical mixing term (VM). Figures are plotted using MATLAB R2014b (http:// www.mathworks.com/).

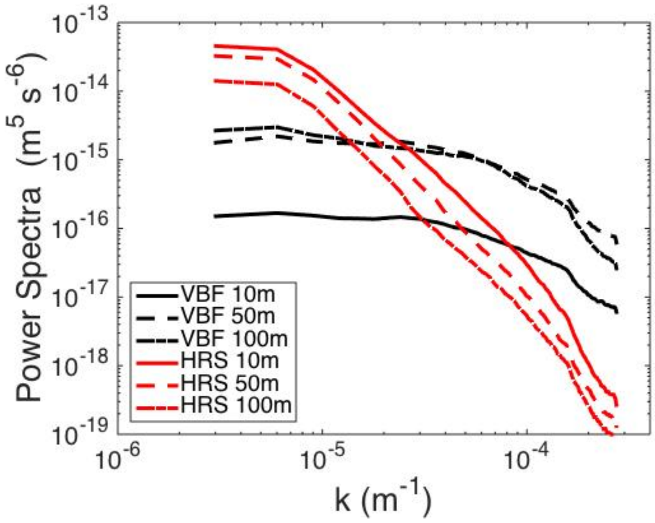

Figure S6 | The monthly mean (in December) power spectra for the vertical buoyancy flux (black lines) and horizontal shear (red lines) at 10m (solid), 50m (dashed) and 100m (doted-dashed) depth as a function of horizontal wavenumber magnitude. Figure is plotted using MATLAB R2014b (http://www.mathworks.com/).
